# Intersecting Vulnerabilities: The Impacts of COVID-19 on the Psycho-emotional Lives of Young People in Low- and Middle-Income Countries

**DOI:** 10.1057/s41287-020-00325-5

**Published:** 2020-11-09

**Authors:** Prerna Banati, Nicola Jones, Sally Youssef

**Affiliations:** 1 UNICEF WCA, Dakar, Senegal; 2 ODI/GAGE, London, UK; 3 GAGE Lebanon, Beirut, Lebanon

**Keywords:** Adolescents, Gender, COVID-19, Psycho-emotional, Vulnerabilities, Mental health, LMIC, I3, I14

## Abstract

Across diverse contexts, emerging evidence suggests that the COVID-19 pandemic is increasing levels of anxiety and stress. In calling for greater attention to people’s psychosocial and emotional well-being, global actors have paid insufficient attention to the realities of the pandemic in low- and middle-income countries, where millions of people are already exposed to intersecting vulnerabilities. Chronic poverty, protracted violence, conflict and displacement, coupled with weak health, education and protection systems, provide the backdrop of many adolescents’ lives. Drawing on qualitative in-country telephone interviews with over 500 adolescents in Ethiopia, Côte d’Ivoire and Lebanon, this article unpacks the age and gendered dimensions of COVID-19 and its response. We conclude by discussing the implications for COVID-19 recovery efforts, arguing that embedding adolescent-centred, inclusive approaches in education, community-based health and social protection responses, has the potential to mitigate the psycho-emotional toll of the pandemic on young people and promote resilience.

## Résumé

De nouvelles données probantes issues de différents contextes suggèrent que la pandémie de COVID-19 augmente le niveau d'anxiété et de stress. En appelant à une plus grande attention au bien-être psychosocial et émotionnel des populations, les acteurs mondiaux ont accordé une attention insuffisante aux réalités de la pandémie dans les pays à revenu faible et intermédiaire, où des millions de personnes sont déjà exposées à des vulnérabilités croisées. La pauvreté chronique, la violence prolongée, les conflits et les déplacements, associés à la faiblesse des systèmes de santé, d'éducation et de protection sociale, sont la toile de fond de la vie de nombreux adolescents et adolescentes. Cet article s'appuie sur des entretiens téléphoniques qualitatifs avec plus de 500 adolescent.e.s et 55 informateurs-trices clés en Éthiopie, en Côte d'Ivoire et au Liban, pour démêler les dimensions sexospécifiques et liées à l’âge de la COVID-19 et de sa réponse. Nous abordons en conclusion ce que cela implique pour les efforts de relance suite à la COVID-19, en faisant valoir que l'intégration d'approches inclusives et centrées sur les adolescent.e.s dans l'éducation, dans les réponses sanitaires et sociales à base communautaire, a le potentiel d'atténuer le bilan psycho-émotionnel de la pandémie sur les jeunes et de promouvoir la résilience.

## Introduction

Worldwide, documented experiences of the COVID-19 pandemic have described elevated levels of anxiety and stress (Salari et al. 2020). During its initial stages, mental health and psychosocial concerns were high among global priorities, prompting recommendations by the World Health Organization (WHO) on how to maintain psychosocial and emotional well-being (WHO 2020). Yet these recommendations have seemingly overlooked the realities of the pandemic in low- and middle-income countries (LMICs) and, in particular, in conflict-affected settings, where many people are already exposed to multiple and intersecting vulnerabilities.

Chronic poverty and subsistence living, protracted violence, and conflict and massive displacement, coupled with weak health, education and protection systems, provide the backdrop of adolescents’ lives in the contrasting settings—Ethiopia, Côte d’Ivoire and Lebanon—that our evidence is drawn from. Using qualitative evidence from virtual research with more than 500 adolescents, we unpack the age and gendered dimensions of the social and economic consequences of the pandemic and its response.

As highlighted in the discussion and conclusions, our broader aim is to contribute to dialogue on how national government and development partners can more effectively intervene to mitigate the psycho-emotional toll of the pandemic and promote resilience among adolescents in some of the most difficult places in the world to be a young person.

## Conceptual Framework

A high prevalence of mental health conditions among people living in crisis—22.1%—has been recently reported in the literature (Charlson et al. 2019). Despite this high burden, relatively little research has unpacked this among adolescents. Notable exceptions exist and include Samuels et al. (2017) who note the need to go beyond the health system to ensure broader well-being for adolescent girls in fragile contexts. The findings highlight the challenges of humanitarian and biomedical models to address such complexities. Newnham et al. (2020) have explored the impact of natural disasters in China and Nepal on psychosocial well-being of adolescents, and have found gendered differences in psychological distress, while also observing post-traumatic growth and strengthened connections with families.

**Fig. 1:**
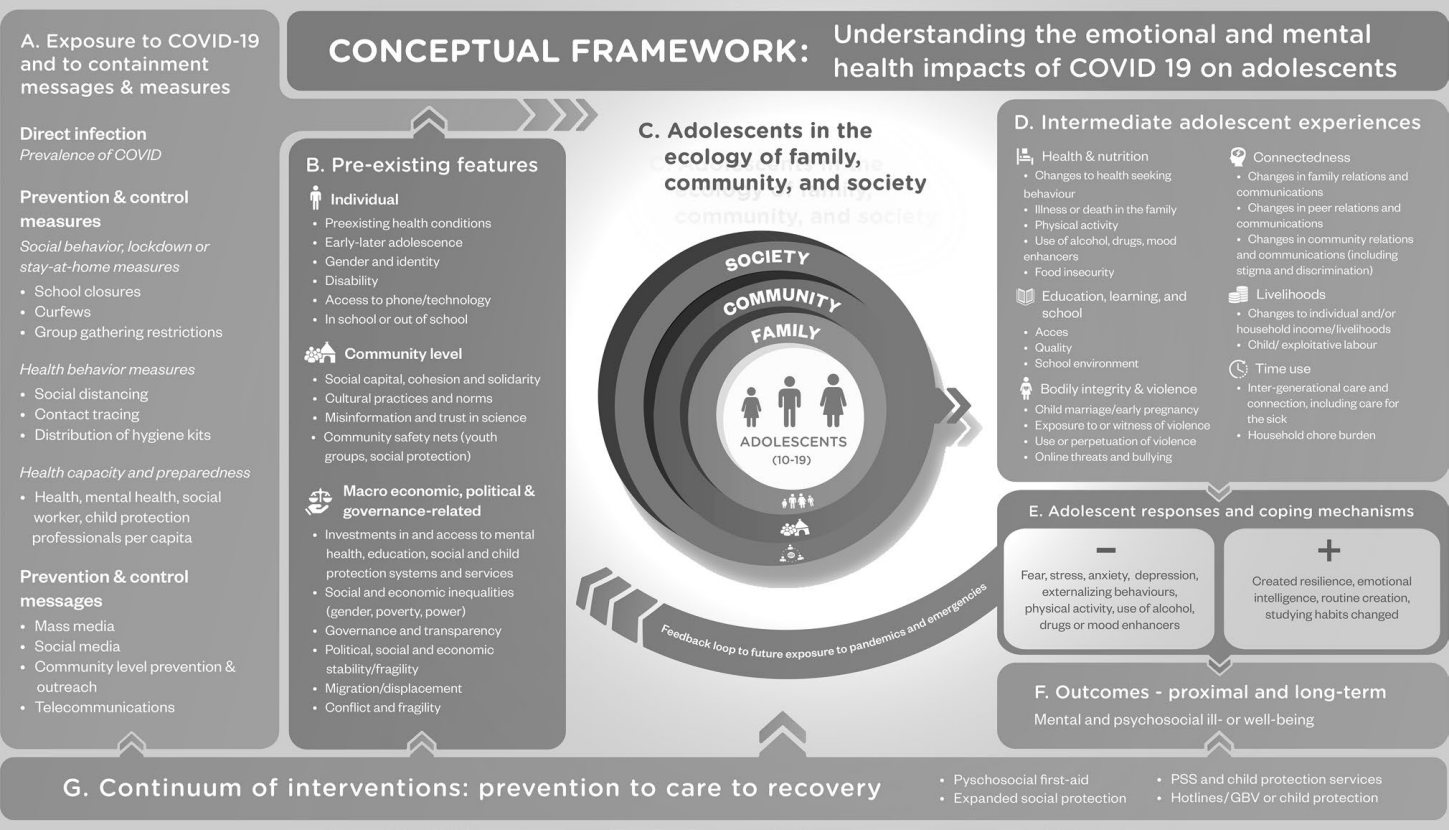
Conceptual framework: understanding the emotional and mental health impacts of COVID-19 on adolescents

Given the relative dearth of research on adolescent mental health and psychosocial well-being in crisis contexts (e.g. Samuels et al. 2017), we draw on the available literature in developing a conceptual framework (see Fig. 1) that explores how social, economic and political determinants as well as individual, family, community and policy responses to the pandemic shape not just adolescent experiences of it but also their coping strategies. This framework guides the analysis of our primary research findings.

Exposure and related measures (Box A) take three forms: (1) the risk of direct infection, which although low among adolescents is not negligible (WHO 2020); (2) prevention and control measures, which have significant implications for young people (e.g. social-behavioural measures such as curfews, health behavioural measures such as hand washing and wearing of masks, and health capacity and preparedness (such as implementation of contact tracing systems); and (3) messaging. Social media messaging may be a particularly effective way of reaching adolescents (Livingstone et al. 2017). These different types of exposures influence adolescent behaviours through diverse pathways that may have multiplier effects. For example, school closures may mean a loss of school meals, contributing to food insecurity and psycho-emotional stress for the adolescent and their parent. At the same time, mass media messaging (on television, for example) may create fear and amplify anxieties.

Pre-existing features (Box B) from the individual through to the macro level shape psychosocial vulnerability and resilience. For example, macro-level investments in psychosocial support and care interventions in schools will have direct impacts on adolescents’ well-being, including reductions in bullying (Ameratunga et al. 2018). At community level, cultural practices and norms can amplify or attenuate the impacts of containment measures. Tightly knit communities, for example, may be able to mitigate the adverse impacts of home containment by providing family-to-family support; conversely, communities already facing social cohesion deficits may become more fractious, exacerbating psychosocial stressors stemming from the pandemic. At the individual level, social identities such as gender and age can determine vulnerability to, and ultimately impact of, COVID-19 exposure and related containment measures (Samuels et al. 2017). Adopting a life-course perspective highlights how determinants operate at every level of development, and the nature of cumulative risks and opportunities (Banati and Lansford 2018). Disability, as well as other intersectionalities, will mean that young people experience control and containment measures differently (Małachowska et al. 2020a).

Our framework is rooted in the seminal work of Bronfenbrenner (1994), situating the adolescent at the centre of a multi-level socio-ecological model (Box C). *At the macro level,* importance is attributed to society, including the broad set of structural, cultural and functional features that impact adolescents directly or via meso-level mediators (e.g. politics, history and economy, environment, social norms, values and beliefs). *At the meso level,* institutions and policy processes (public and private) at national and community levels constitute important dimensions. *At the micro level,* the interpersonal and family environment play important roles.

*Power* operates at all levels to create societal inequities, including in the case of gender hierarchies and interpersonal violence (Davies et al. 2019). *Gender* is closely linked to the distribution of power, and emerging evidence suggests that females are disproportionately impacted by COVID-19 prevention measures that limit access to services, employment and freedoms. They also carry the additional care and domestic work burden brought by periods of quarantine at home (Camilletti et al. 2018). Exposure to gender-based violence and child marriage—already high in many LMICs—may also increase due to school closures (Kanem 2020; Banati and Camilletti 2018). Gender norms can also be reinforced or exacerbated during a crisis (Morgan and Davies 2020; Hupkau and Petrongolo 2020).

Intermediate adolescent experiences (Box D) are organised across six domains: health and nutrition, education, bodily integrity and violence, connectedness, livelihoods, and time use. For example, closure of schools worldwide and shifts to online education in some countries have impacted learning (Azevedo et al. 2020). Given the role of school environments in shaping adolescent development, the pandemic is likely to have impacts in this regard too (McNeely et al. 2002)—impacts that may be greater in low-income settings, where children often rely on school for basic needs such as meals.

Adolescent responses and coping strategies (Box E, connected through a reciprocal arrow with Box D) describes the interdependence between coping strategies and lived experiences. There is evidence that adolescents with positive family relationships use more active coping strategies at home and school (Zimmer-Gembeck and Locke 2007; Stavropoulou and Jones 2013). However, there is less evidence on the success of these coping strategies in moderating adolescent stress (Elgar et al. 2003; Yoo 2019). Positive responses/coping could include ‘created resilience’, which results from successful adaptation and coping through adversity. Negative outcomes include internalising behaviours (such as depression and anxiety), which disproportionately impact adolescent girls; and externalising behaviours (such as violence or aggression), which disproportionately impact adolescent boys. Both negative and positive responses/coping (Box E) can contribute to either resilience or vulnerability, hence the feedback loop linking Box E with Box B.

The framework also captures the proximal and longer-term mental ill-health or well-being outcomes (Box F), while Box G describes the continuum of interventions (prevention, care and recovery), which intervenes across the framework. Examples include psychosocial first aid or child protection services, which we discuss in the concluding section.

## Research Methods

The paper draws primarily on virtual qualitative research to explore the consequences of the COVID-19 pandemic for the most disadvantaged households, and coping strategies used by adolescent girls and boys to respond to vulnerabilities. Our sample is presented in Table 1. While we acknowledge differences in the samples, combined data from the three countries offer important perspectives from diverse geographical and governance contexts about young people’s experiences of the early onset effects of the pandemic when data generation was challenging.

**Table 1:** Research sample

	Sample size	Locations	Dates when data collected	Method of data collection
Ethiopia	119 adolescents aged 15–19 years	6 communities (urban: Dire Dawa city and Ebenat town, South Gondar; rural: South Gondar, East Hararghe, and Zone 5, Afar)	Round 1: April 2020 Round 2: May 2020	Phone interviews (30–45 min)
Côte d’Ivoire	349 adolescents aged 10 to 19 163 female, 186 male	10 communities (Port-Bouet, Marcory, Treichville, Cocody, Adjamé, Abobo, Yopougon, Bingerville, Kaniasso and Bondoukou)	May–June 2020	Phone interviews; some in-person interviews
Lebanon	100 adolescents aged 15–19 years 50 female, (including 25 married girls) 50 male	Ein Al-Helwei, Wavel Palestinian refugee camps, Syrian collective shelters and host communities, Beqaa Valley	Round 1: April, Round 2: May/June	Phone/WhatsApp Interviews (30–90 min)

Data collection took place in Ethiopia and Lebanon during two rounds of telephone interviews with adolescents in April and May 2020, conducted by in-country researchers who had a pre-existing relationship with respondents through the Gender and Adolescence: Global Evidence (GAGE) longitudinal research study (see Małachowska et al. 2020b). The adolescents were selected to ensure representation from diverse geographic locations (urban/rural/camp) and social groups (in education/out-of-education; married; with disabilities; refugees). Ethics approval was secured from the Overseas Development Institute Research Ethics Committee (for the Ethiopia and Lebanon samples), and in Ethiopia, from the regional research ethics boards of the Ministry of Health.

Data on Côte d’Ivoire is drawn from qualitative interviews conducted by UNICEF in May and June (2020) with young people representing diverse geographic and social groups as part of adaptation for COVID-19 programming and in consultation with government authorities. Ethics standards met UNICEF’s ethics for evidence generation protocols and approval was granted by the Government to undertake this work as part of UNICEF’s broader in-country data collection and programming processes.

Analysis of the interviews drew on detailed debriefings with the country research teams, followed by thematic analysis of the transcripts aligned with the conceptual framework discussed above.

## Findings

In discussing our findings, we emphasise adolescents’ specific experiences and responses to the pandemic and government mitigation measures (i.e., Boxes D and E from Fig. 1).

## Contexts in Which Adolescents were Living Prior to COVID-19

The three study countries vary widely in terms of population, human development indicators, multi-dimensional poverty and connectivity.^1^ However, all three are characterised by conservative gender norms and high rates of gender inequality, as well as very high levels of state fragility (see Table 2).

**Table 2:** Country overview

	Pop. (million)	Human Development Index (HDI) value and ranking (2019)	Multidimensional Poverty Index (MPI) % of population	Gender Social Norms Index	Gender Inequality Index	State fragility index score and rank	Level of connectivity: percentage of population with internet
Ethiopia	109	0.47 (173rd)	83.5	85	0.508 (123rd)	94.2 (23rd)	18.6
Côte d’Ivoire	25	0.52 (165th)	46.1	n/a	0.657 (157th)	92.1 (29th)	43.8
Lebanon	6.85	0.73 (93rd)	n/a	96	0.362 (79th)	85 (44th)	78.2

*Source* UNDP (2019) and Fund for Peace (2019)

Within these contexts, under-investments in education and health services, the justice sector and social protection systems are reflected in poor well-being outcomes across the life course (Patton et al. 2016). Adolescents face multiple vulnerabilities across a range of domains, including sexual and reproductive health (SRH), education, violence, child marriage, access to decent work, and poverty (Table 3).

**Table 3:** Adolescent vulnerabilities

	Adolescent fertility rate in 2018 (World Bank 2020)	Complete lower secondary school in 2018 (UNESCO 2020)	Physical violence at home/school	Exploitative work	Child poverty	Child marriage
Ethiopia	66.7	21% f 22% m	49% of adolescents aged 11–17 experience violence at home; 30% at school (ACPF 2014)	49% of adolescents 15–17 and 46% of girls involved in child labour (UNICEF 2019)	88% of children experience at least one dimension of multidimensional poverty (CSA and UNICEF 2018)	40% by age 18 (CSA and ICF 2016)
Côte d’Ivoire	117.6	22% f 36% m	65% have experienced physical violence in last month (INS and ICF^2016^	22% of adolescents 15–17 engaged in hazardous work in last week—24% boys, 19% girls (INS and ICF 2016)	59% of adolescents are poor (Humanium, n.d.,b)	27% by age 18 (INS and ICF 2016)
Lebanon	14.5	54.3% f 55.6% m	13% experience severe physical punishment by parents; 25% experience violence at schools	7% of adolescents work (Humanium, n.d., Lebanon)	No child poverty data available; 27% of population are at national poverty line	6% by age 18

UNESCO figures are not available for recent years. These are UNDP figures for people aged 25 years and over with at least some secondary schooling

## Exposure to COVID-19 and to Containment Measures and Messaging

As of July 2020, the three countries have relatively low but steadily increasing infection and death rates. Côte d’Ivoire has a strict curfew in place and non-essential shops and schools remain closed, whereas Ethiopia and Lebanon had initial lockdowns and curbs on mobility that have since been relaxed. In Lebanon, schools have reopened but in Ethiopia they are only reopening in October 2020 (see Table 4).

**Table 4:** COVID-19 infection caseload and government responses

	Caseload (as of 2 July 2020)	Deaths (as of 2 July 2020)	Government lockdown/curfew	School closures
Ethiopia	5846	103	Temporary lockdown and initial restrictions on travel, but since lifted. State of emergency in place until August 2020	Closed since late March
Côte d’Ivoire	9702	68	Curfew from 9 p.m. to 5 a.m., movement restrictions from capital to countryside; non-essential shops closed	Closed since 16 March
Lebanon	1796	5	Strict lockdown with curfew from mid-March to end April, 5-phase reopening with restrictions largely lifted by June 8	Closed since 29 Feb, resumed online from end May

Adolescents in our sample reported mixed exposure and responses to public health messaging. There were significant urban–rural divides in knowledge about transmission mechanisms and how to prevent the infection spreading, and higher levels of knowledge among boys compared to girls in Ethiopia and Côte d’Ivoire, though not in Lebanon. Gender differences were largely due to the gender digital divide: boys (more likely to be in work and have access to cash) have greater access to personal phones and internet connectivity, while girls reported limited connectivity due to conservative gender norms and surveillance of girls’ use of devices by fathers and older brothers in particular. Adolescents also reported that older family members tended to dominate access to TV and radio sources of information.

Urban and in-school adolescents emphasised that young people generally complied with hygiene and social distancing measures. However, many also highlighted that behaviour change in their communities was uneven. Among rural respondents and those out of school, adolescents tended to have poorer awareness about COVID-19 and mitigation measures, and even when they were aware, the guidance was often not practical to their context. For example, in drought-stricken rural communities in Ethiopia, the emphasis on hand washing was dismissed as impractical without external support. Other young people noted that they were relying on their faith to protect them. Indeed, some actively resisted social distancing when it hindered their religious practice, especially during holidays such as Orthodox Easter or Ramadan.

## Adolescent Experiences and Responses to COVID-19

Beyond social distancing and hygiene measures, adolescents highlighted a range of impacts of the pandemic on their lives, many of which compound pre-existing disadvantages rooted in poverty, conservative gender norms, disability or refugee status. We discuss similarities and differences in adolescent experiences across the three focal countries, as well as the coping strategies employed by adolescents during the early period of the pandemic.

### Health and Nutrition

#### Health-Seeking Behaviour

Our findings show that few young people or their families had tested positive for COVID-19; however, those that have contracted it (particularly adolescent girls and young women) faced stigma from their families, colleagues and community. As one respondent in Côte d’Ivoire said:

After my recovery [from COVID-19], I started to have conflicts with my sister who came to live with us because she had just given birth. She saw me as a reservoir of contamination for her child, and she even decided that I would not go back to work, and my parents validated her decision.

Adolescents discussed other changes in their health-seeking behaviours. In Lebanon, because healthcare is expensive and inaccessible for disadvantaged households who lack health insurance (Hanna-Amodio 2020), many young people were worried about access to care and medicines should they or their families contract COVID-19, especially adolescents whose family members have underlying health conditions. As a 19-year-old Palestinian girl explained:

Both my parents are sick and need medication. My father is disabled… How will we pay for all of this if we cannot find money to buy food? I do not even have a way to escape… I just go up to the roof and cry every day.

In camp settings, these concerns are magnified due to lack of access to healthcare and overcrowded living conditions. A 19-year-old Palestinian girl explained:

It would be disastrous if the virus spread… The camp is crowded and small, and if someone is infected, all the people in the camp will die… No one cares or will help us…

Health-related fears are also amplified by adolescents’ inability to follow guidance on preventing the spread of COVID-19 due to financial challenges. As a 17-year-old Syrian boy stated:

I can’t buy a face mask … The cost … used to be 2,000 [Lebanese pounds (1.32 USD], now the cost is 20,000 [13.20 USD]. We cannot afford bread to eat, how can we buy face masks?

In Côte d’Ivoire and Ethiopia, young people highlighted pre-existing barriers to healthcare and medicines (see Jones et al. 2020), especially financial constraints, a dearth of youth-friendly service providers, distance to clinics and hospitals, and a tendency to rely on traditional sources of support. They added that concerns about infection were a major deterrent to health-seeking behaviour, especially for sexual and reproductive healthcare. Adolescents and key informants in Afar, Ethiopia, noted that married girls were fearful of seeking antenatal care and opting for home-based birth instead, though several maternal deaths had been reported as a result. Our findings in Côte d’Ivoire also point to a rise in adolescent pregnancies, probably due to decreased access to contraceptives given disruptions to clinics and outreach services.

#### Food Consumption and Nutrition

Our findings underscored that the economic consequences of lockdowns and restrictions on movement had reduced household incomes, often leading to greater food insecurity. In Lebanon, most respondents expressed concerns over their households’ inability to buy enough food due to loss of income and price increases. Reflecting the severity of the deteriorating situation among refugee and host communities in Lebanon, a common refrain from adolescents was: *‘Dying from the virus is better than dying from hunger’.* Refugees and married adolescents appear to be especially vulnerable. As a 17-year-old married Syrian girl noted:

The situation is miserable here… We are not eating, we started baking bread in the camp because we cannot afford it anymore. It is very hard and tiring… I feel I am suffocating under all the pressures.

Supplies for menstrual management were also a concern among refugee girls in Lebanon. Adolescent girls reported either resorting to borrowing from local stores or using pieces of cloth at home. As an 18-year-old Syrian mother noted:

I stopped buying sanitary pads and started using whatever cloths I have at home to be able to buy diapers for my son and I try to use only one or two pieces in the day… I am so tired of life, I wish I could sleep forever and never wake up in the morning.

Adolescents in Ethiopia expressed similar concerns. Curbs on transport between urban and rural areas are leading to food shortages, with prices escalating rapidly. As an 18-year-old male student from South Gondar noted:

We are facing huge cost increases of food items… sorghum, rice… due to the restriction on public transportation. We can even face more serious challenges if the lockdown continues this way.

Adolescents from internally displaced households in East Hararghe noted that because they had already stopped receiving social protection support and were facing food shortages prior to the pandemic, they were concerned that if markets continued to be blocked, they would face serious hunger problems.

### Education, Learning and School

For adolescents in school prior to the pandemic, the closure of educational institutions due to COVID-19 has significantly disrupted their schooling. Many have limited or no access to online learning, while others, especially in Ethiopia, reporting having been compelled to drop out of school permanently.

In Lebanon, the education system is characterised by weak public provision and reliance on high-cost private institutions, contributing to one of the highest levels of educational inequality in the region (Chaaban and el Khoury 2016). The shift to distance learning has exacerbated these inequalities, as some schools have adopted online distance learning during lockdown while others are providing none. Power shortages and energy costs from private providers hinder access to the basic infrastructure needed for distance learning (El Turk and Cherney 2016). An 18-year-old Palestinian girl reported:

I stopped studying because we don’t have internet, we don’t have money to pay for it… The connectivity is very bad and the electricity always cuts, which make it impossible to attend the classes.

Some adolescents also had difficulties adapting to the online teaching methods and reported lack of support from teachers. A 15-year-old Palestinian girl explained:

This education method has caused us severe stress and depression. We are given more lessons and homework than we usually get at school without explaining them to us and … with little support from our teachers.

Some adolescent students also expressed strong fears and worries about dropping out of school, especially in the context of the worsening economic climate, as underscored by this 19-year-old Palestinian girl:

We are not studying now and I do not know if I will ever be able to study again. I already had to stop for two years because my family did not have the money and it has been already hard for me to pay the fees this year, and now our situation is even worse.

In Ethiopia, while there have been some efforts at online schooling in large urban centres, this was not accessible to the adolescents in our sample, who explained that with national exams looming, closure of schools was a particular source of stress. Others noted that education had become deprioritised given the pressing economic challenges their communities were now facing. As an 18-year-old adolescent from East Hararghe noted:

There is no education now… My friends and I spend much of our time in watering the khat plants [a stimulant] and we go home only in the evening. We have no time to study. We also do not know how long the schools will be closed. Now no one talks about education here. We talk only about our farms and the impact of coronavirus on our economy.

In Côte d’Ivoire, learning was also significantly impacted by school closures. Many adolescents in our sample reported feeling demotivated in their studies and training, and worried about access to food following school canteen closures. Income-earning activities took priority over alternative/distance learning.

### Bodily Integrity and Violence

Young people in our sample highlighted that during the pandemic they were being exposed to heightened bodily integrity risks, including increased exposure to intra-household violence, mixed exposure to child marriage risks (depending on context), and elevated community-level violence (in the case of Lebanon and, to some extent, Côte d’Ivoire).

Across contexts adolescent girls (but not boys)^2^ reported heightened exposure to intra-household tensions and violence. In Côte d’Ivoire, girls reported an increase in physical and psychological violence, especially due to household size increases as shelter orders were put in place. In Lebanon, since lockdown, girls from host and refugee communities alike reported an increase in intra-family tensions and problems, partly due to the whole family being confined together at home and increasing stresses over economic hardships. As a 15-year-old Palestinian girl reported:

My family started skipping many of our needs to be able to pay the rent and the internet fees. We are skipping a lot of food due to increasing prices. We have a lot of fights and problems…

Similarly, a 16-year-old Lebanese girl noted:

The tension has increased dramatically… We are all at home suffering from a bad psychological state. My father is very nervous, and we are all nervous and sad, we try to hide the nervousness but it shows on our faces. The reason… is the worry about tomorrow and surviving.

Married Syrian girls also reported increased tensions with their husbands, in-laws and neighbours, and reports of intimate partner violence were also noted.

Married girls in Ethiopia reported similar household concerns about financial fragility and intimate partner violence. As a 17-year-old girl in Afar stated:

I woke up early to prepare food for our children. After… I woke him [my husband] to keep our younger child [1 year old] until I got back from fetching water, he became irritated… He shouted at me, picked up the stick near him and hit me across my head, I don’t know what happened then…

It is also important to note that some of the intra-household violence reported was perpetrated by adolescents, particularly adolescent mothers, due to increased economic, domestic and childcare pressures. As a 17-year-old Syrian mother commented:

I am always nervous, shouting at the children. They used to play outside, but now they are with me all the time while I am working and baking. I am not able to handle all of this and sometimes, when I get nervous, I lash out at my children, hitting them to relieve my anger.

#### Child Marriage

Our findings on risks of child marriage were mixed. In Lebanon, while two of the adolescent Palestinian girls in our sample had become engaged during lockdown, other adolescent girls were of the view that the economic crisis will result in a lower marriage rate. They explained that they will have to postpone marriage as they are not able to afford the increasing costs of marriage and house items. As a 19-year-old Palestinian girl said: *Now that everything is expensive, there will be no grooms as the boys will not be able to afford marriage*.

Lebanese girls believe the economic crisis will result in fewer marriages among Lebanese girls but more among Syrian girls, as marriage to a Syrian girl is considered cheaper. As this 17-year-old Lebanese girl explained:

The spinsterhood is increasing among the Lebanese girls because the [Lebanese] men or young males prefer to marry Syrian girls because her dowry is not high…

Palestinian and Lebanese boys in our sample believe that the economic situation has made it difficult for them to think about marriage and to have hopes for the future. As an 18-year-old Lebanese boy explained: *I cannot think of having a future anymore, I cannot think of marriage or having a house. It is over for us, we do not have a future.*

By contrast, in Ethiopia, adolescent girls and boys both highlighted that they were under increasing pressure to marry now that they were out of school, especially because school closure coincided with the traditional wedding season in three of the six communities where our research was carried out. Adolescents reported that parents pressured daughters, even in early adolescence, to marry, given that school is out—not least because the limited presence of local authority officials and teachers (many of whom have returned to their home towns) makes it easier to do so. (Teachers in particular have been instrumental in reporting impending child marriages.) As a 17-year-old adolescent boy from South Gondar noted:

Since Easter is a marriage season, people have been married off since then. There are girls who were married off at the age of 15. Most of them are from rural parts. There is an 8^th^ grader who got married from the town, but it was her decision though.

Similarly, in Afar, a 17-year-old girl explained that:

For girls it is better the school is opened because the school is quite important for us, not only to learn… but it also helps us escape from arranged marriage.

Our findings also showed that some (albeit relatively few) boys who were previously attending secondary school in neighbouring towns were being pressured to marry rather than *‘sit idle’* at home. This was striking given that families had already invested in their sons’ education for a significant number of years. It underscores the extent to which households are already having to resort to negative coping strategies as household incomes contract, and lose confidence in their children being able to complete their studies, especially with school closures resulting in a repeat of the current academic year.

#### Community-Level Violence

Our findings also point to an increase in some forms of community-level violence due to the pandemic. In Côte d’Ivoire, some respondents noted that there was considerable stigma associated with contracting COVID-19, and those who had were fearful of community reactions.

Interviews with Syrian boys also underscored fraying social cohesion with the host community in Lebanon and increased fears of violence. Syrian boys who live in collective shelters are becoming more confined to their homes due to increasing fears of being targeted by the Lebanese authorities and community. As a 16-year-old Syrian refugee boy reported:

We cannot go out or visit our relatives and friends because our neighbours are scared that we have corona[virus]. When they see us outside they call the police, they are annoying us a lot. We stay at home and we close the windows so that they would not see us.

Fears about increased crime levels were also reported by some Lebanese boys, which translated into greater anxiety and some resorting to weapon carrying.

Given the wider socio-political and economic environment in Lebanon, and increasing strains on social cohesion, adolescents (especially refugee adolescents) expressed fears and anxiety about their future in the country. An 18-year-old Syrian boy stated:

We are heading towards an unknown path, we cannot turn back and we will die in both cases – either from the virus or from hunger, if the lockdown continues.

### Connectedness

#### Changes in Relationships with Family

Young people in our sample highlighted that relationships with family members were being reshaped by added pressures to contribute domestic or agricultural labour, especially since the closure of schools. In rural communities in Ethiopia, boys emphasised that they were expected to do agricultural work for many hours without pay. As a 15-year-old from South Gondar explained:

I would not have been employed in another household if school had not been closed… but since school is closed now, my mother made me work in another household to herd cattle and she promised me to return me back to school when it opens.

In Lebanon, girls (especially married girls) underscored that while domestic and care work responsibilities existed pre-COVID-19, they had been heightened during the pandemic—often to an intolerable extent. A 16-year-old Syrian girl emphasised:

I cannot tolerate it anymore, I feel I am suffocating. My in-laws keep fighting with me, their demands never end … My husband never defends me because he does not want to have problems with his family.

#### Changes in Relationships with Peers

Our findings here were mixed, highlighting that due to conservative gender norms and strong restrictions on girls’ mobility even before the pandemic, adolescent girls have been particularly affected. In Lebanon, for example, during lockdown Lebanese and Palestinian girls’ mobility has been further restricted, ruling out altogether the few activities they were allowed to do outside. A 19-year-old Palestinian young woman explained:

We feel sad and oppressed at home. It is true that we were not doing anything big with our lives, but we were doing something, at least we were able to visit our friends and our neighbours.

The physical distance from friends and limited privacy at home has also resulted in loss of peer-support networks, especially for girls, who typically lack access to digital devices and privacy at home. A 19-year-old Palestinian refugee from Syria stated:

I do not have a phone and I am barely allowed to use my mom’s phone. I cannot talk to my friend when I am feeling sad. Also I cannot talk about my problems while I am at home and everyone is around me.

School closures have also impacted girls’ access to their peer-support networks, as socialising at school away from the surveillance of the family used to provide them with space that cannot be always matched online. A 15-year-old Lebanese girl explained:

I stopped talking to my friends because I do not like to talk to them on the phone. It was different at school, I used to see my friends and confide in my best friend…. now I do not see anyone and I feel sad at home.

In Ethiopia, while similar themes of social isolation and separation from peers due to school closures emerged among adolescent girls, boys highlighted that they were largely continuing their social interaction with peers, especially in communities where *khat*-chewing ceremonies are a regular part of daily life. Adolescents explained that in Dire Dawa and East Hararghe, even when most food markets had been closed, *khat* markets were not (some young people thought this was because the government feared public unrest if they closed them), and regular *khat*-chewing sessions with neighbours and friends persisted.

#### Changes in Relationships Within Communities

In Ethiopia and Côte d’Ivoire, young people did not highlight major changes in their relationship with others in their community, but in Lebanon—perhaps because of the pre-existing economic and political crisis that had led to uprisings in late 2019/early 2020—shifts in relationships between refugee and host communities emerged as a key concern among Syrian refugee adolescents.

A 17-year-old married Syrian girl reported:

I went to the local market next to us and a Lebanese woman asked me to stay away from her: ‘You Syrians are infected with the virus.’ I was upset but I could not do anything. She started asking me to leave the shop…

### Livelihoods

Economic stresses emerged as a very significant source of anxiety across all three countries, especially for adolescent boys and married adolescent girls. In Lebanon, stresses over economic hardships are amplified for working boys, especially Syrian boys (who are often the family breadwinners). Many noted that they were already resorting to borrowing money for food and rent. Boys who live in group shelters reported having to move during lockdown as they were unable to pay the rent. An 18-year-old Syrian young man stated:

The conditions are especially difficult during this period of time [the lockdown]… I am the main breadwinner in my family and I am not working and we do not have money. I borrow money when I can to get food for my family and on top of everything we were evicted from our house, we moved to a small house with two rooms only and we are 11 members in my family.

Many married Syrian girls expressed high levels of distress about the fact that their husbands’ source of income had been cut during the pandemic. Some living in informal tented settlements explained that they have continued to work in agriculture, but doing fewer hours (in the early morning) out of fear of being caught by the authorities and penalised; they did so to maintain an income for their households as the men were out of work during lockdown.

In Ethiopia, young people in urban areas emphasised how the restrictions on transport between urban and rural areas were constraining income-generation opportunities, including small trade and daily construction work. Girls employed in the hospitality industry and who had migrated from rural areas reported that they had no savings or networks to fall back on, and were extremely worried about how they would meet their basic subsistence costs. As a 17-year-old waitress noted:

I do not know how I am going to pay rent. It has been more than two weeks since I stopped working and we do not know when [restaurants] will open up. I do not know what to do.

#### Risky and Exploitative Work

As a result of the economic hardships facing families and communities during lockdown, a number of young people reported that they were being compelled to engage in—or at least consider—risky and exploitative work. In Lebanon, some adolescent boys noted that economic hardships and the stresses these put on working boys could also push them into drug dealing and cultivation, especially in the northern Beqaa regions, where cannabis cultivation and drug trafficking are common. An 18-year-old Lebanese adolescent boy explained:

I used to work in spring and summer, but now everything is closed and there is no work. I live with my mother and we are not able to pay rent, internet fees and buy food. Is this a living?… I never thought of dealing drugs before, but now I find myself thinking of it… I feel desperate.

In Ethiopia, some young people also noted that adolescent girls in towns and cities are especially vulnerable to sexual harassment and abuse while working. As an 18-year-old young man from South Gondar explained, because of the closure of bars, many people are turning to locally brewed alcohol and traditional alcohol houses, and adolescent girls helping their mothers find themselves exposed to unwanted sexual attention:

Some girls also support their mothers in making and selling ‘tella’ [a local alcoholic drink]… Selling tella can expose the girls to sexual abuse…

#### Access to Social Protection

While there has been much discussion at the global level about expanding social assistance as a mitigation measure against the economic fallout of COVID-19, very few of the respondents in our sample had access to social protection support in the immediate aftermath of the pandemic. In Côte d’Ivoire, analysis of vulnerable households showed that beyond the widespread slowdown of income-generating activities, there was a scarcity of social transfers to offset their increased fragility. Young people reported that an increase in household debts—especially by women—was common, representing either contract debts in kind with local shopkeepers or the exhaustion of savings and social capital. Similarly, in Lebanon, refugees noted that they lacked humanitarian assistance in general, and that none had been forthcoming since the pandemic. As an 18-year-old married Syrian girl explained:

We do not receive any aid from the UN [UNHCR] and my husband is not working now… How will we buy food with these prices?

While Ethiopia’s flagship Productive Safety Net Programme (PSNP) reaches more than 8 million rural and urban households, few adolescents in our sample belonged to beneficiary households. In urban areas, adolescent migrants who were often living without family support underscored that they had no access to social protection. This was particularly challenging for girls who had migrated to take on domestic work, and young people with disabilities in our sample who, due to stigma and discrimination, often reported a lack of family support. For example, an 18-year-old adolescent with a visual impairment explained his concerns:

I don’t have any support from my family, I have been living with the 350 birr [9.47 USD] stipend I am paid from our school and from selling napkins, mobile cards… But amid the pandemic, I stopped my business. I am forced to return to my family in a rural area, but there is no public transport and so I’m very worried about the coming days and how I will survive.

### Time Use

A key theme that emerged across all three countries was changes in adolescents’ time use. Girls, and married girls in particular, complained of heightened time poverty, with parents (and husbands and in-laws) expecting them to do many hours of domestic and care work, with little time for relaxation or recreation. A 17-year-old Syrian refugee girl in Lebanon explained:

There is a lot of tension at home, all men are sitting at home without work and we are all nervous and fighting all the time. The men’s demands never end and they release their anger at us, even though we are working outside and doing everything at home while they sit all day without doing anything.

The additional domestic responsibilities that girls bear at home, especially during COVID, has also contributed to reduced time to stay in contact with peers. A 19-year-old Palestinian young woman noted:

I rarely talk to my friends now. I feel there is no time at home and I do not have the time to talk to them. I wake up late, pray, clean the house, prepare food and I feel the day has finished.

While, as discussed earlier, Ethiopian boys in rural areas reported that they were facing strong pressures to undertake long hours of agricultural work daily, they still recognised that the situation was particularly challenging for girls, who lacked any recreation time. As an 18-year-old young man from South Gondar (Ethiopia) observed:

Both boys and girls have their own tasks to do…. To be honest, girls are more engaged in their tasks, as the boys at least have time to entertain; playing table football, and drinking tella [local alcohol].

## Adolescent Coping Strategies

Adolescents’ coping strategies in these contexts have varied, with our respondents providing evidence of both negative and positive strategies. In terms of negative coping, a number of adolescents (especially in Lebanon where the pandemic has exacerbated a severe economic and political crisis), reported experiencing serious depression and several even voiced suicidal ideation. Others talked about escapism. Several adolescent boys in Ethiopia and Lebanon reported resorting to alcohol or drugs to escape the pressures of the pandemic, noting that this was widespread among their peers. Fatalistic thinking and turning to religion were also common reactions across all three contexts.

However, our findings also underscore that some young people had adopted positive coping strategies, and learning from these cases can reveal potential entry points to enhance young people’s resilience in crisis contexts. In Ethiopia, some young people highlighted that they were volunteering to distribute hand sanitiser and food packages in their neighbourhoods and were actively sharing public health messaging about social distancing among their social networks, either in person or through social media. In Lebanon, while a number of adolescent boys noted that they were spending long hours on online games, others reported playing more football in their neighbourhood to release stress and had even reduced their smoking habits—in part because financial pressures meant they could no longer afford cigarettes and hookah pipes. Some adolescent girls reported having taken up new hobbies such as photography, meditation or dance in order to improve their mood and well-being. As an 18-year-old Lebanese young woman explained:

Corona[virus]'s time made us explore our talents. I started to meditate by watching the sky and photographing it… When I started taking these pictures, it gave me a feeling of comfort as it is a beautiful view.

## Conclusions and Policy Implications

This paper has highlighted significant multidimensional, gender- and age-specific psycho-emotional effects of the COVID-19 pandemic and government containment measures on adolescents in three diverse LMICs. Our findings show that the pandemic and the public health response to it are exacerbating pre-existing age vulnerabilities for adolescents, especially in terms of anxiety and stress, and limited access to youth-friendly health services, education services during school closures and peer networks. Our data has also underscored intersecting gender-specific vulnerabilities including reduced access to menstrual hygiene supplies as family incomes contract, intensified time poverty for adolescent girls during school closures and lockdowns due to the unequal intra-household gender division of labour (and especially for married girls), as well as heightened exposure to sexual and gender-based violence risks. In some communities, girls (and to a lesser extent boys) are also at heightened risk of child marriage given the economic consequences of the pandemic, whilst adolescent boys are at greater risk of exploitative forms of labour. The paper has also revealed both common coping strategies (e.g. relying on faith, volunteering, escapism) as well as divergent strategies employed by adolescent girls (e.g. taking up new hobbies) and boys (e.g. playing online games, substance abuse).

These findings suggest that inclusive, adolescent-centred approaches need to be considered in COVID-19 recovery efforts to ‘build back better’, and that interventions will need to be adapted to the varying intensity and duration of the pandemic and diverse containment measures in place in different contexts. By focusing on high-impact strategies, efficiencies can be gained which will be important in the coming period of shrinking fiscal space anticipated in LMICs. Recognising that the evidence here has captured the short-term impacts on adolescent realities, and that follow-up social research will be needed to ascertain the patterning and extent of more enduring, longer-term psycho-emotional consequences, we propose four potential responses.

1. *Leveraging the education response* as schools in more than 100 countries begin to reopen offers an important entry-point to integrate gender- and age-responsive psychosocial support into existing curricula. Evidence demonstrates the effectiveness of school-based approaches to addressing adolescent mental health, with success in low-income contexts (Shinde et al. 2018). The education sector has also adapted and responded to COVID-19 through new models of delivery, including virtual schooling and radio schools in resource-constrained settings, which would also allow for the integration of psychosocial modules and referrals to promote adolescents’ access to support services. Combined with in-person mentoring (with social distancing), this could give young people the personal interaction they need with a competent non-family adult—something which the evidence suggests can play an important role in fostering adolescent well-being (Scales and Roehlkepartain 2018).
2. *The expansion of COVID-related social protection in LMICs* provides a unique opportunity to identify and support the most vulnerable groups, including adolescents with disabilities or married adolescents. There is significant potential for social protection systems to promote adolescent well-being, gender equality and transformative change as a core pre-condition for long-term and sustainable poverty reduction (Holmes and Jones 2013). Evidence also suggests that cash transfers can result in improved mental health outcomes among young people aged 15–24, with significant reductions in depressive symptoms (Kilburn et al. 2014).
3. *Community-based delivery models adapted to local population and cultural needs* could also be more fully harnessed in the provision of mental health services for adolescents. As our findings have highlighted, cultural and religious attributes of illness and belief influence how people seek help, and drive behaviour change and access to services as well as outcomes for mental health. Community-based models need to engage holistically with the adolescent experience, recognising the damaging consequences of stigma and discrimination (Betancourt et al. 2015). Human-centred design approaches and youth- or peer-led interventions show early promise. However, many integrated community-based approaches have fallen short of the minimum standards required for adequate provision of mental health support, including referral services, and especially with regard to interventions to prevent and respond to sexual and gender-based violence, including those addressing child marriage. As such, it will be important to focus on addressing barriers that impede adoelscent girls’ access to health and other services by considering mobility constraints, opening hours, access to female staff and service providers, alongside safety concerns, childcare responsibilities, and social distancing restrictions (UNHCR 2020). To unlock the potential of these approaches, constraints in the enabling environment will also need to be addressed, including engagement of religious leaders, addressing stigma, fake news and misinformation.
4. *The expansion of inclusive virtual platforms provides an opportunity to expand psychosocial first aid delivery for adolescents*, particularly in urban settings with good internet connectivity (Kauer et al. 2014), but also in hard-to-reach communities where health infrastructure may not be readily available. These approaches have been studied in conflict- and disaster-affected populations—for example, among children affected by the East Asian tsunami (Pairojkul et al. 2010). Evidence suggests that digital tools offer several advantages—not least that services can be provided at scale, at low cost and largely in private, reducing stigma associated with seeking help. However, further efforts are needed to determine how digital programmes that deliver prevention and promote positive mental health reach adolescents from all parts of society, including the more vulnerable, and how digital infrastructure can be expanded without exacerbating digital inequalities.

## Footnotes

1 Connectivity in the Palestinian camps is likely to be lower due to poverty, but disaggregated figures are not available.

2 We hypothesise this is due to both lower exposure as well as lower reporting on the part of boys due to dominant cultural norms around masculinity, and will follow up on this in subsequent research.
